# Hospital Meetings

**Published:** 1904-08-06

**Authors:** 


### HOSPITAL MEETINGS.

### EAST LONDON HOSPITAL FOR CHILDREN.

The half-yearly Court of Governors of this hospital was held on Thursday, 28th ult., Colonel Charles Needham presiding.

The Chairman said it was with much regret that the board of management were again compelled to call the attention of governors to the finances of the hospital, which were at the present time a source of considerable anxiety. Although the total receipts for the six months ended June 30th last exceeded those for the corresponding period of 1903 by nearly £600, this total fell short of the ordinary maintenance expenses, and had necessitated a further loan from the bankers. The total deficit of the hospital and seaside branch now stood at £2,474 13s. 3d., £2,000 of which was represented by the loan from the bank, the remainder being made up of debts to tradesmen. The payment of interest on the loan was an unsatisfactory item of expenditure, and one which it was particularly desirable to avoid. To meet it a portion of the invested property might be sold; but, apart from the reduction of permanent income involved, the securities would, in the present state of the stock market, have to be disposed of at a considerable loss, and the board could only recommend their sale as a last resource. That considerable efforts had been made to curtail expenditure as far as was compatible with efficient working was shown by the fact that while there had been an actual increase in the work, both at Shadwell and at Bognor, the expenditure was less, by about £222, than in the corresponding period of 1903. The public, the chairman remarked, were tired of charitable entertainments, and it was therefore a matter for congratulation that the fête recently held at the Royal Botanical Gardens had resulted in a sum nearer £400 than £300. They were much indebted also to Lady Clarke Jervoise for a cheque for £175, the proceeds of a matinée at the New Theatre organised by her. In order to help the funds, an interesting little book, called "The Story of an East London Hospital," published 20 years ago, containing a history of the foundation of the hospital by Mrs. Heckford, had been republished in an attractive form, and was issued at half a crown. Under extraordinary expenditure was included the cost of providing outside blinds to the Ward Pavilion (£89), extra equipment for the electrical department (£50), and £265 for alterations in the theatre, including the introduction of an improved system of heating and ventilating, and the installation of electricity for lighting and motor purposes. Mention had already been made, in the annual report, of the necessity for erecting whooping cough wards, and the reconstruction of the very inadequate isolation accommodation. It was found that the cost of these works would amount to £10,000, so the Board decided not to commit themselves to so large an expenditure at once, and entered into a contract, for the erection of the whooping cough wards only, for £6,090. Good progress was being made, and the building would probably be completed before the end of the year, when it was the intention of the Board to use the new wards for the general infectious cases arising in the hospital, while the contractors were engaged in reconstructing the isolation block. The Council of King Edward's Fund had made grants amounting to £3,350 towards these works, the last grant being coupled with the condition that operations should be begun in the present year. The Worshipful Company of Drapers had since voted a grant of £500 for the same purpose, conditionally upon the total amount required (£10,000) being obtained within a reasonable time. He therefore most earnestly asked for contributions, in order that this generous offer might be taken advantage of. Commenting on the financial position of the hospital, Colonel Needham said the expenditure was £10,000, while invested funds did not yield £1,000, so that for the remaining £9,000 the hospital depended upon the generosity of the public. Whether the public would continue to produce this sum was, in his opinion, a very doubtful question. If not, the wards would have to be closed, or securities sold out, which, as already mentioned, was highly undesirable. If the public did not come to the rescue, he saw no possibility of carrying on the work for another two years, and he was afraid it would result in what a great many business men had declared to be inevitable, viz., that the hospitals of this city must go upon the rates. Whether that would be satisfactory he could not say. But at any rate a slur would be involved if hospitals managed for so many years by private individuals were thrown upon the public. That, however, was inevitable unless more financial support were forthcoming.

Mr. H. Luttman Johnson, in seconding the adoption of the report, said if saving were to be effected, it should be in the out-patient department; this was a question that was receiving attention all over London.

The report was adopted, and the meeting concluded with a vote of thanks to the chair.

### ROYAL ORTHOPÆDIC HOSPITAL.

The 65th Annual Court of Governors of the Royal Orthopædic Hospital was held, by kind permission of the National Orthopædic Hospital, at 234 Great Portland Street, W., on Friday, 29th ult., Mr. Walter Emden presiding. The chairman was supported by Sir Ernest Flower, M.P., Mr. Gordon Campbell, and the secretary, Mr. Tate S. Mansford.

In giving a general review of the report, the last to be presented before the completion of the amalgamation of this hospital with the National Orthopædic Hospital, the chairman said that although there had been a slight falling off in subscriptions, which were only £418 as against £440 in the previous year, and the donations were also slightly lower, yet the total income amounted to £3,291. This was partly made up of a legacy of £1,591, the amount from this source in the previous year being only £100, while there was an excess of income over expenditure of £943 17s. 11d. It should be added that contributions amounting to nearly £1,000 were received for the rebuilding fund, and this no doubt affected in some measure the general income receipts. The expenditure for the past year was £2,347, compared with £2,494 in 1902, showing a substantial reduction. The expenditure having already been brought down to the lowest point consistent with due efficiency, this reduction would not have been possible had it not been for the removal to the present temporary premises, in anticipation of which the expense of repairs and cleaning was naturally kept down, and other economies effected. It was necessary to sell £1,658 of stock in order to reduce the heavy liabilities carried forward from the previous year. The committee had hoped to still further reduce the debt, which at the beginning of the year amounted to £2,158 13s. 2d., but owing to the falling off in subscriptions and donations their hope was not realised, and the year ended with a debt of £1,438 10s. 7d.

It must be realised, he said, that small hospitals, working separately for the same object, must necessarily be more expensive than large ones, while there was not the same encouragement to the public to support them. The past year had been a most eventful one in the long history of the hospital. The negotiations for amalgamation with the National Orthopædic Hospital, which were communicated to the Governors by circular on June 26th last, and authorised at the special court on July 8th, had now been brought to a satisfactory conclusion, and the committee were pleased to report that the draft charter and laws for the future government of the combined institutions, to be known as the Royal National Orthopædic Hospital, now only awaited the formal approval of the Privy Council. As soon as the Royal Charter of Incorporation was granted, the amalgamation would be completed. He regretted that efforts to induce the City Orthopædic Hospital to join in the scheme had not been successful, but in his opinion it was only a question of time, since, so far as could be seen, there was nothing to be gained by standing out, whereas by combining, administrative expenses could be cut down. It was a question of supplying a larger number of beds and having more funds to lay out on developments, while a large institution undoubtedly commanded a greater degree of respect from the public, and was, in consequence, better supported. The committee were happy to report that they had been released from the agreement to take the site in Coram Street, Bloomsbury, on which it was originally intended to rebuild the hospital, and they desired to record their obligations to His Grace the Duke of Bedford for the ready and generous manner in which he relieved the hospital from an obligation which might have been a serious obstacle to the negotiations for amalgamation. A considerable sum had been expended on the works, amounting to over £2,000, but, in accordance with a resolution of King Edward's Hospital Fund for London, this amount had now been reimbursed to the hospital by that fund, so that no loss would be suffered by the hospital in consequence of the abandonment of the original rebuilding scheme.

Mr. Gordon Campbell having seconded the adoption of the report, Sir Ernest Flower warmly supported the policy of the committee, and commended the combined institution to the public. He thought the governors were aware that it was intended to rebuild a very large portion of the premises in Great Portland Street, indeed nearly all except the part in which the meeting was being held. The cost would, no doubt, be considerable, but there would be a handsome sum from the disposal of the property in Hanover Square and Oxford Street, and the Committee hoped that when they came before the public, their appeal would meet with a generous response. They were exceedingly pleased with the response to the very short-lived appeal for funds to rebuild in Bloomsbury, and he thought they might conclude that the scheme so successfully brought to a close would have the warm approval of a number of generous friends. He joined with the Chairman in his regret that the City Orthopædic Hospital had not fallen in with the scheme. It was not, however, too late, and he might speak for both the combined institutions in saying that nothing would give them greater pleasure than to welcome amalgamation with that hospital. In the multiplication of special hospitals there lay a waste of energy and a tendency to extravagant administration, and he believed one great central orthopædic hospital for London was very desirable. He hoped that next year there would be a large attendance at the combined court of the two hospitals so happily amalgamated.

The report was adopted, and honorary officers elected for the ensuing year. The court then became special, and resolutions were passed approving of the disposal of the former site and empowering the committee to affix the hospital seal to the agreement. The rules for the new institution (the Royal National Orthopædic Hospital), as compiled by the joint committee of both institutions, were received and adopted.

A vote of thanks to the chairman concluded the meeting, at which several gentlemen interested in the hospital, but not governors, were present.

